# Rhinovirus and Innate Immune Function of Airway Epithelium

**DOI:** 10.3389/fcimb.2020.00277

**Published:** 2020-06-19

**Authors:** Haleh Ganjian, Charu Rajput, Manal Elzoheiry, Umadevi Sajjan

**Affiliations:** ^1^Department of Thoracic Medicine and Surgery, Lewis Katz Medical School, Temple University, Philadelphia, PA, United States; ^2^Department of Physiology, Lewis Katz Medical School, Temple University, Philadelphia, PA, United States

**Keywords:** dsRNA, rhinovirus, antiviral responses, ER stress, autophagy, COPD, asthma

## Abstract

Airway epithelial cells, which lines the respiratory mucosa is in direct contact with the environment. Airway epithelial cells are the primary target for rhinovirus and other inhaled pathogens. In response to rhinovirus infection, airway epithelial cells mount both pro-inflammatory responses and antiviral innate immune responses to clear the virus efficiently. Some of the antiviral responses include the expression of IFNs, endoplasmic reticulum stress induced unfolded protein response and autophagy. Airway epithelial cells also recruits other innate immune cells to establish antiviral state and resolve the inflammation in the lungs. In patients with chronic lung disease, these responses may be either defective or induced in excess leading to deficient clearing of virus and sustained inflammation. In this review, we will discuss the mechanisms underlying antiviral innate immunity and the dysregulation of some of these mechanisms in patients with chronic lung diseases.

## Introduction

Healthy adults inhale about 7–8 L of air per minute or ~11 L of air per day. Therefore, the lung is by far the largest organ in humans that is in direct contact with the environment. Along with air, we also inhale microorganisms and environmental pollutants; therefore, the lung is equipped with several powerful defense mechanisms. Conductive airways, which act as a passageway for the air to move in and out of the lungs, moisten, equilibrate the temperature, filter the inhaled air, and protect the alveoli, which participates in gas exchange. The epithelium that lines the conductive airways, in addition to providing a physical barrier to separate the environment from the lung milieu, also plays a pivotal role in shaping the lung innate and adaptive immunity. Basal cells in the airway epithelium have wound healing properties, so that the airway epithelium repair and regenerate rapidly after the injury. Besides, airway epithelial cells also communicate with other innate immune cells, such as macrophages, neutrophils and dendritic cells, to promote pathogen clearance and limit lung inflammation. Therefore, the airway epithelium through its barrier and innate immune functions play an essential role in clearing the inhaled pathogens and harmful pollutants to maintain homeostasis in the lung.

Patients with chronic lung disorders such as chronic obstructive pulmonary disease (COPD), asthma and cystic fibrosis often show structurally and functionally altered airway epithelium. Such changes may affect the responses to respiratory infections, sometimes accelerating the progression of lung disease in these patients. This review will focus on the innate immune responses of airway epithelium to rhinovirus, which causes common colds in healthy subjects but exacerbates lung disease in patients with COPD, asthma and cystic fibrosis. We will also review the dysregulation of antiviral immunity of airway epithelial cells in patients with chronic lung disorders.

## Conductive Airway Epithelium

**Figure 1:**
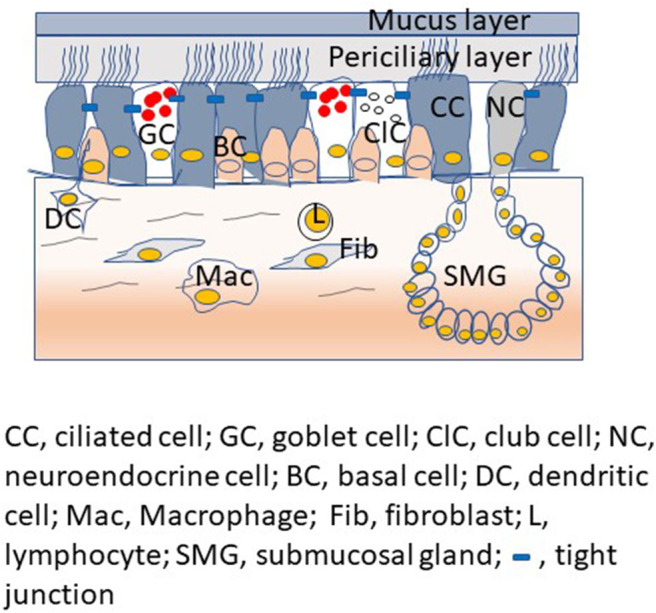
Proximal airway mucosa showing different type of airway epithelial cells with airway surface liquid, submucosal gland and professional innate immune cells in the submucosa.

The proximal conductive airways show pseudostratified morphology with different cell types (Figure 1). The luminal cells, which are in direct contact with the environment, include ciliated cells, mucus-secreting goblet cells, and club cells, another type of secretory cell. Basal cells are located under the luminal cells and closer to the basement membrane. Basal cells are specialized airway stem cells with a capacity to regenerate fully functional airway epithelium following injury (Rock et al., 2009). There are also submucosal glands in the proximal airways, which secrete mucus and water into the airway epithelial surface. In contrast, airway epithelium in the distal conductive airways is cuboidal and primarily consists of ciliated cells with few club cells and airway basal cells. Small airways are devoid of goblet cells and submucosal glands.

Airway epithelium in the proximal airways is overlaid by airway surface fluid, which consists of a thin layer of periciliary less viscous liquid and a layer of semi-viscous mucous. The secretions of club cells and submucosal serous cells contribute to the periciliary fluid. On the other hand, the gel-forming mucins, MUC5AC and MUC5B expressed by goblet cells and the mucosal cells of submucosal glands contribute to the mucus layer (Ostedgaard et al., 2017). A recent study suggests that club cells may also be one of the sources for MUC5B in the proximal and distal airways (Okuda et al., 2019). Height and the viscosity of the periciliary fluid are critical factors as they determine the efficient unidirectional beating of cilia. The mucus layer, which is semi-viscous, traps the inhaled pathogens and other particulate materials and the cilia. Subsecuently, the mucus layer with its unidirectional movement sweep the trapped material toward pharynx for removal from the respiratory tract (Knowles and Boucher, 2002). There are also cell surface tethered mucin glycoproteins that interact with glycosaminoglycan keratin sulfate and this complex is assumed to act as a periciliary brush, creating another layer of protection and preventing the penetration of particulates and microorganisms (Button et al., 2012; Kesimer et al., 2013). The airway epithelial surface liquid also consists of a mixture of host defense molecules such as nitric oxide, lactoferrin, lysozyme, β-defensins, and cathelicidins. These molecules are secreted by airway epithelium, submucosal glands and other innate immune cells of the lungs (Singh et al., 2000; Ganz, 2002; Do et al., 2008). These host defense molecules are capable of killing bacteria, viruses and fungi directly or by limiting essential micronutrients required for successful colonization of pathogens; thus, these defense molecules function as a chemical shield against inhaled pathogens (Zasloff, 2002; Brogden, 2005). Furthermore, the tight and adherence junctional complexes located at the apicolateral border of airway epithelial cells also contribute to defense function by preventing paracellular invasion of inhaled pathogens. Paracellular permeability of airway epithelium is maintained through the cooperation of two mutually exclusive tight and adherence junctions. Tight junctions are located apically in the intercellular junctional complexes. These junctions inhibit water and solute flow, and regulate ion movement through the paracellular spaces (Schneeberger and Lynch, 1992; Pohl et al., 2009; Ganesan et al., 2013). Tight junctions also separate the apical from the basolateral cell surface domains to establish cell polarity (Shin et al., 2006). Adherence junctions mediate cell-to-cell adhesion and promote the formation of tight junctions. These intercellular junctions also prevent the inhaled microbes and allergens from crossing the airway epithelial barrier via paracellular route and activate professional innate immune cells present in the submucosa. Besides, intercellular junctions serve as platforms that regulate gene expression, cell proliferation, and differentiation (Balda and Matter, 2009; Koch and Nusrat, 2009).

In addition to acting as a physical barrier, airway epithelial cells actively participate in recognition of pathogens by various pattern recognition receptors (PRR), which are divided into transmembrane receptors and those localize to intracellular compartments. While the transmembrane receptors include Toll-like receptors (Mayer et al., 2007) and C-type lectins (Gavino et al., 2005; Heyl et al., 2014), the intracellular receptors include NOD like receptors (NLR) (Slevogt et al., 2007; Hirota et al., 2012; He et al., 2016; Han et al., 2019), RIG-I and MDA5 (Wang et al., 2009; Slater et al., 2010). Recognition of pathogen associated molecular patterns by PRR activates signal transduction pathways leading to the expression of antimicrobial factors such as defensins, cathelicidin, nitric oxide, interferons, and interferon-inducible factors (Vareille et al., 2011; Ganesan et al., 2013; Denney and Ho, 2018). These antimicrobial factors can kill the infecting pathogens directly without the involvement of other innate immune cells of the lungs. The airway epithelial cells upon recognition of pathogens also express pro-inflammatory chemokines and cytokines to recruit other innate immune cells to the airways to enhance elimination of the pathogens and resolve inflammation.

Airway epithelial cells being at the interface of the environment and the lung milieu are always in contact with the inhaled microbes and microbial products, and this may cause persistent inflammation of respiratory tissue even if the inhaled substance is not harmful. Since inflammation can cause tissue damage, frequent and persistent inflammatory responses may lead to airway remodeling and tissue destruction. To overcome this effect, airway epithelial cells also express intrinsic anti-inflammatory molecules such as inhibitory arachidonic acid metabolites, which include lipoxins, resolvins, and prostaglandin E2 (Bonnans et al., 2007; Levy and Serhan, 2014; Colby et al., 2016), suppressor of cytokine signaling (SOCS)1 and 3 (Niwa et al., 2018; Xander et al., 2019), anti-inflammatory cytokines (IL-10 and TGF-β), soluble cytokine receptors/receptor antagonists (sIL-1RN, sIL-13RA2, sTNFR2, IL-1RA), protease inhibitors (SLP1, SERPINA1, SERPINB1, TIMP-1), and immunosupressive cell surface molecules (B7-H1, B7-DC, IL-13RA2, and FASL) (Levine et al., 1997; Hamann et al., 1998; Kim et al., 2005; Matsumura et al., 2007). Many of these molecules are induced by pro-inflammatory cytokines and Th2 cytokines, suggesting that they may be regulated by negative feedback pathways to dampen the inflammation. Therefore, dysregulation or imbalance of inflammatory to anti-inflammatory signaling can result in persistent or overt lung inflammation, ultimately leading to irreversible tissue damage.

There is also ample evidence that airway epithelial cells communicate with other innate immune cells via secreted molecules to clear pathogens and maintain homeostasis. Under unstimulated conditions, bronchial epithelial cells secret transforming growth factor (TGF)-β, which in turn (a) inhibits LPS-induced TNF-α in monocytes and macrophages, (b) induces expression of IL-10 and arginase-1 in dendritic cells, an indication of alternative activation, and (c) inhibits the proliferation of T cells (Mayer et al., 2008). Bronchial epithelial cell expression of programmed death receptor-ligand1 (PD-L1) in response to respiratory syncytial virus activates and promotes proliferation and antiviral function of CD8+ve T cells in a co-culture model of bronchial epithelial cells and CD8+ve T cells (Telcian et al., 2011). In a co-culture model of bronchial epithelial cells and dendritic cells, epithelial cells secretes GM-CSF, GCSF, and VEGF in response to respiratory virus infection and this enhances type I IFN expression in plasmacytoid dendritic cells that is critical for viral clearance (Rahmatpanah et al., 2019). Under an allergic environment, airway epithelial cells express thymic stromal lymphopoietin (TSLP) and IL-33 in response to viral infection and promote generation of TH2 cells (Mehta et al., 2016). Airway epithelial cells express TSLP, IL-33, and IL-25 in response to LPS and house dust mite allergen and cause dendritic cell activation and allergic inflammation (Hammad et al., 2009). These findings indicate that the interactions of airway epithelial cells with other innate immune cells plays a critical role in shaping the overall local innate immunity in the lungs.

## Rhinovirus

Rhinoviruses (RVs) belong to the *Picornaviridae* family and are responsible for the majority of the common colds (Makela et al., 1998). In patients with underlying lung disease such as COPD, asthma, and cystic fibrosis, RV exacerbates the respiratory illness (Lee et al., 2012; Etherington et al., 2014; George et al., 2014; Stolz et al., 2019). RVs are small (27 to 30 nm), non-enveloped viruses with an icosahedral capsid and a single-stranded positive-sense RNA (+ssRNA) genome made up of 7,200 bases (Palmenberg and Gern, 2015). So far, more than160 serotypes of RVs have been discovered. They have been divided based on phylogeny into three species, Rhinovirus-A (RV-A), Rhinovirus-B (RV-B), and Rhinovirus-C (RV-C). Whereas RV-A and RV-B have smoother, spherical capsid structure, RV-C has 60 dominant spike-like protrusions, or “fingers,” on the outer surface of the virion (Liu et al., 2016). RV-C has a large deletion in VP1, one of the structural proteins, and it lacks a protruding “plateau” around each of the 5-fold vertices, a characteristic feature of RV-A and RV-B (Basta et al., 2014; Liu et al., 2016).

RV-As include 80 serotypes, RV-Bs include 32 serotypes, and the recently found RV-C species contains at least 57 serotypes (http://www.picornaviridae.com). All three RV subgroups bind to plasma membrane glycoproteins to gain entry into the cells. Historically, RV-A and -B strains are classified into two groups depending on their cellular receptor utilization for internalization into the cells. Approximately 89 serotypes of RV-A and -B species belong to the major group and bind to human ICAM-1 (Greve et al., 1989); thus, showing the species specificity. The minor group RVs, which consist of at least 12 serotypes of RV-A, bind to the low-density lipoprotein receptor (LDLR) family with no species specificity. The receptor binding sites were mapped around the 5-fold axis of symmetry in viral capsid, but they have also shown to be different between the major and minor group RVs. The first domain of ICAM-1 binds the virus inside the canyon (a 2.5 nm depression) surrounding the dome at the vertex (Kolatkar et al., 1999). In contrast, the LDLR ligand-binding domain, composed of multiple ligand-binding repeats, attaches to the top of the star-like surface-exposed structure at the vertex (Hewat et al., 2000). These binding interactions are required for entry into the host cells by endocytosis. In addition to these receptors, RV-A and RV-B also interact with TLR2 on airway epithelial cells and macrophages, and this interaction modulates RV-induced innate immune responses (Unger et al., 2012; Ganesan et al., 2016; Bentley et al., 2019; Xander et al., 2019). Binding to TLR2 may not be necessary for viral entry into the host cells. More recently discovered RV-C binds to CDHR3, a member of the cadherin family of transmembrane proteins, which mediates RV-C entry into host cells. An asthma-related mutation (Cys529 → Tyr) in CDHR3 is associated with increased viral binding and progeny yields *in vitro*, and it is proposed to be due to increased cell surface expression of this receptor (Bonnelykke et al., 2014; Bochkov et al., 2015). Further, the extracellular domains 1-3 of CDHR3 was shown to mediate RV-C interaction with host cells (Watters and Palmenberg, 2018). However, the mechanisms of entry or replication of RV-C using CDHR3 are so far unknown.

The RV genome encodes four structural proteins, VP1, VP2, VP3, and VP4, which make up the viral capsid, and seven non-structural proteins, 2A, 2B, 2C, 3A, 3B, 3C, and 3D, which participate in RV genome replication and polypeptide processing. The viral life cycle involves viral capsid binding and entry into the airway epithelial cell, the release of viral genome into the cytoplasm, transcription and translation of the viral genome, polypeptide processing and subsequently virion assembly. The VP1 structural protein mediates viral binding to host cell surface (Palmenberg and Gern, 2015). RV-A and RV-B binding to their receptors initiates the process of receptor mediated endocytosis, which allow particle entry into the host cell and trigger genome uncoating within the endosome (Fuchs and Blaas, 2010). These receptors direct virus uptake via clathrin-dependent or independent endocytosis or via macropinocytosis (Fuchs and Blaas, 2010; Blaas, 2016). Acidic pH in the endosomes triggers uncoating of the virus and release of viral RNA into the cytoplasm. The viral genome is transcribed by viral 3D RNA polymerase. The genome is translated with host factors, and the polypeptide is subsequently processed by viral 2A and 3C proteases (Fuchs and Blaas, 2010). This is followed by virion assembly, genome packaging, and virus shedding, either by lytic or non-lytic processes. Not much is known about the replication cycle of RV-C.

## Innate Immune Responses of Airway Epithelial Cells to Rhinovirus Infection

As discussed above, airway epithelial cells express cell surface and cytoplasmic PRRs. Amongst these PRRs, TLR2 is expressed on the cell surface, TLR3 and TLR7 in the endosomes and MDA5 and RIG-I in the cytoplasm. These PRRs are implicated in RV-induced innate immune responses in airway epithelial cells.

### Replication-Dependent and Independent Pro-inflammatory Responses

**Figure 2:**
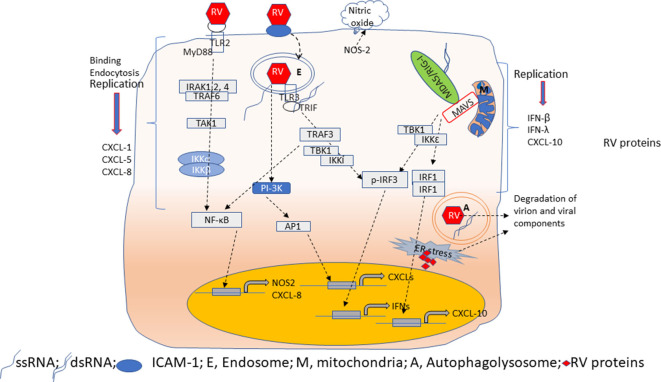
Overview of innate immune responses of airway epithelial cells to RV. Airway epithelial cells show replication-independent CXCL responses which occurs as a result of activation PI-3 kinase/AP1 following binding and endocytosis of RV. Interaction of RV with TLR2 may induce CXCL-8 and NOS-2 expression via MyD88 signaling pathway. NOS-2 generates nitric oxide by catalysis of arginine. CXCL responses are also elicited when TLR3 binds to RV dsRNA via NF-κB. In contrast, RV-stimulated IFN and CXCL-10 responses are primarily due to dsRNA generated during RV replication. Binding of dsRNA to TLR3 induces IFNs via TBK1/IKKi/IRF3 activation. On the other hand, cytoplasmic receptors MDA5 or RIG-I upon binding to dsRNA translocate and interact with MAVS expressed on mitochondria. This interaction triggers activation of TBK1/IKKε to induce expression of IFNs and CXCL-10 via IRF3 and IRF1/NF-κB, respectively. During viral replication, accumulation of RV proteins and RV genome activates ER stress and induces autophagy. These processes degrade RV proteins to limit virion assembly.

RV primarily infects airway epithelial cells. RV binds to both ciliated and non-ciliated cells (Jakiela et al., 2008; Lopez-Souza et al., 2009; Faris et al., 2016; Griggs et al., 2017; Tan et al., 2018; Warner et al., 2019) in the airway epithelium. RV infection causes very little cellular damage suggesting that immediate innate defense mechanisms of airway epithelial cells can efficiently inhibit viral replication; thus, establishing an antiviral state. The airway epithelial cells express chemokines such as CXCL-1, CXCL-5, and IL-8 in response to RV interaction with its glycoprotein receptors and viral endocytosis (Newcomb et al., 2005; Sajjan et al., 2006; Bentley et al., 2007). Inhibition of phosphoinositide (PI)-3 kinase or TLR2 reduces RV-induced CXCL-8 expression (Newcomb et al., 2005; Bentley et al., 2007; Unger et al., 2012) (Figure 2). Interaction of viral genome with endosomal TLR7, which recognizes single-stranded RNA, may also induce pro-inflammatory cytokines (Triantafilou et al., 2011; Tan et al., 2018). At later stages of infection, expression of these chemokines is also stimulated via activation of TLR3, MDA5 and RIG-I signaling by dsRNA, an intermediate generated during viral replication (Hewson et al., 2005; Sajjan et al., 2006; Slater et al., 2010; Triantafilou et al., 2011). Airway epithelial cells also express and secrete growth factors, such as amphiregulin, epithelial cell growth factor (EGF) and epiregulin all of which activates EGF receptor signaling to increase IL-8 and ICAM-1 levels. Inhibition of EGF receptor, Erk1/2 or STATs attenuates RV-induced IL-8 in the airway epithelial cells (Liu et al., 2008). Upregulation of CXCL-8 and granulocyte colony stimulating factor was also observed in the nasal secretions of healthy human volunteers after the experimental infection with RV (Grunberg et al., 1997; Gern et al., 2000), and these responses correlated with neutrophil infiltration in the nasal cavity (Grunberg et al., 1997). Even though these early pro-inflammatory responses are necessary, they must be tightly regulated to prevent excessive inflammation and subsequent tissue damage. Dual specificity phosphatases, which inhibit various mitogen-activated protein kinase (MAPK), toll-interacting protein (Tollip), a negative regulator of TLR signaling pathway, and TLR2-activation mediated IRAK-1 degradation within a short period after RV infection may all contribute to limiting RV-induced inflammation (Unger et al., 2012; Manley et al., 2019; Dakhama et al., 2020).

### Replication-Dependent Responses

Airway epithelial cells express CXCL-10/IP-10, and type I and type III interferons (IFNs), such as IFN-β, IFN-λ1, λ2, and λ3 in response to replication- efficient RV infection (Figure 2). CXCL-10 recruits activated type 1 T lymphocytes and natural killer cells to the site of infection, which can recognize and kill the infected cells. IFNs, in addition to amplifying type I and III IFN responses, also stimulate expression of interferon-incuced genes such as viperin, MX-1, ISG-15, and double-stranded (ds)-RNA receptors (TLR3, MDA-5, and RIG-I) to establish a local antiviral state. Expression of CXCL-10 and antiviral IFNs in airway epithelial cells is stimulated primarily by dsRNA intermediate generated during RV replication; thus, replication-deficient RV does not stimulate these responses (Wang et al., 2009; Schneider et al., 2010). Airway epithelial cells constitutively express TLR3 in the endosomes; therefore, TLR3 may participate in the early detection of viral dsRNA leading to IRF3 and NF-κB activation followed by expression of CXCL-10 and IFNs (Wang et al., 2009; Slater et al., 2010). Cytoplasmic ds-RNA receptors, MDA5 and RIG-I are IFN-inducible genes. In airway epithelial cells, while RIG-I is constitutively expressed, MDA5 is expressed only after RV infection (Wang et al., 2009 #51; Gimenes-Junior et al., 2019a #21). Interaction of these receptors with viral dsRNA in the cytoplasm enhances expression of IFNs (Wang et al., 2009; Slater et al., 2010; Zaheer et al., 2014). Although RV-stimulated expression of both CXCL-10 and IFNs are induced by activation of dsRNA receptors, CXCL-10 expression is regulated by IRF-1, and NF-κB, but not IRF3 (Spurrell et al., 2005; Zaheer and Proud, 2010). In contrast, the dsRNA-induced expression of IFNs is regulated primarily by IRF3. Both MDA5 and RIG-I are helicases with the CARD domain and interact with the CARD domain of MAVS after recognition of dsRNA in the cytoplasm. This interaction activates IKKα and IKKε to stimulate the expression of CXCL-10 and IFNs espression, respectively, but the underlying reasons for this redundancy are not well-known. Since viral dsRNA is longer and MDA5 recognizes high molecular weight or long dsRNA, it is conceivable that MDA5 may play a significant role in stimulating IFN responses to RV infection. Consistent with this notion, we demonstrated that knockdown of MDA5, but not RIG-I significantly reduced RV-induced IFNs (Wang et al., 2009 #51). In support of our findings, a child with inherited MDA5 deficiency was found to have recurrent RV infection (Lamborn et al., 2017). Nasal epithelial cells from this patient showed an increase in viral transcripts compared to her parents' nasal epithelial cells when challenged with RV. Such an increase in viral transcripts was not observed when the patient's nasal cells were infected with RV. These observations indicate that MDA5 may play a pivotal role in controlling the RV replication in airway epithelial cells via the induction of IFNs.

In response to RV infection, although airway epithelial cells express both type I and type III interferons, the expression of type III IFNs such as IFN-λ1 (IL-29) and IFN-λ2 (IL-28A) is more robust. IFN-λs are expressed at both mRNA and protein levels (Schneider et al., 2010; Chattoraj et al., 2011). While receptors for type I IFNs are expressed in a variety of cell types, type III IFN receptors are expressed only on epithelial cells (Mordstein et al., 2008; Sommereyns et al., 2008). Mice lacking type III IFN receptor show greater susceptibility to influenza A virus, influenza B virus, respiratory syncytial virus, human metapneumovirus, and coronavirus (Mordstein et al., 2010). Therefore, it is conceivable that type III IFNs may also be essential for controlling viral replication in RV-infected airway epithelial cells. Airway epithelial cells express several IFN-inducible genes (ISG) following RV infection (Proud et al., 2008). So far, only viperin and ISG-15 have been investigated for their innate immune properties in the context of RV infection. Whereas knockdown of viperin increased viral load, knockdown of ISG-15 enhanced CXCL-10 production without affecting viral titer in RV-infected airway epithelial cells (Proud et al., 2008; Zaheer et al., 2014), indicating a role of ISGs in inhibiting viral replication and modulation of innate immunity.

### Oxidative Stress and Innate Immunity

Another arm of innate immunity in airway epithelial cells is oxidative stress. Airway epithelial cells in response to RV infection express nitric oxide synthase−2 (NOS-2) *in vitro* and in subjects experiencing symptomatic colds (Sanders et al., 2001). NOS-2 generates nitric oxide, a potent antiviral agent. Human volunteers showed increase in nitric oxide generation in their nasal cavities after experimental infection with RV (Sanders et al., 2004). Exhaled nitric oxide correlated inversely with viral titer at 4 days post-RV infection in these volunteers. Later, nitric oxide was demonstrated to negatively regulate RV-induced CXCL-10 via NF-kB and IRF-1 downregulation (Spurrell et al., 2005; Koetzler et al., 2009; Zaheer et al., 2009); thus, implicating immunomodulatory role for nitric oxide in addition to antiviral property. Kaul et al. reported that replication-deficient RV induces reactive oxygen species via p47-phox while neutralization of reactive oxygen species reduced RV-stimulated IL-8 in these cells (Kaul et al., 2000). We demonstrated that RV transiently disrupts barrier function in polarized and mucociliary-differentiated airway epithelial cells *in vitro* and in a mouse model of RV infection *in vivo* (Sajjan et al., 2008). The disruption of barrier function was dependent on RV-induced oxidative stress via NADPH oxidase (NOX)1 and Nod-like receptor (NLR)X-1 (Comstock et al., 2011; Unger et al., 2014). Airway epithelial cells, which are already under oxidative stress due to preexisting conditions such as bacterial infection or persistent epithelial to mesenchymal transition show reduced IFN expression and higher viral loads in response to RV infection (Chattoraj et al., 2011; Yang et al., 2017). Further, induction of oxidative stress by pretreatment with hydrogen peroxide also attenuated antiviral responses in airway epithelial cells obtained from patients with asthma and COPD, but not healthy subjects. This is because the normal cells express antioxidant genes, such as superoxide dismutase 1 and 2 (Menzel et al., 2019). Our own studies indicate that pretreatment with antioxidant, diphenylene iodonium (DPI) suppresses RV-induced IFN responses in cells from healthy subjects (Sajjan et al., unpublished data). More recently, nasal epithelial cells were demonstrated to express higher IFNs than bronchial cells in response to RV infection, and this was due to increased expression of NRF2-mediated antioxidant response in bronchial epithelial cells (Mihaylova et al., 2018). Conversely, treatment of epithelial cells which are already under oxidative stress with DPI or MITOTEMPO increased IFN responses to normal levels and reduced the viral titers following RV infection (Chattoraj et al., 2011; Gimenes-Junior et al., 2019b). Reduced expression of IFNs in cells under oxidative stress is due to the unavailability of MAVS to the dsRNA receptor. These observations suggest that while induction of oxidative stress during viral infection improves antiviral responses and modulate innate immune responses, preexisting oxidative stress may reduce the antiviral IFN responses.

### Endoplasmic Reticulum Stress in Innate Immunity

The endoplasmic reticulum (ER) supports various processes in the eukaryotic cells. ER membrane provides ample surface area for cellular reactions such as the production of proteins, steroids and lipids, as well as protein folding and secretion. During cell proliferation and differentiation, the ER may be overloaded by molecular chaperones, folding enzymes and massive protein products causing ER stress. Furthermore, pathophysiological stress signals such as viral infection, accumulation of misfolded proteins, hypoxia ER-Ca^2^ depletion can cause ER stress (Kaufman, 1999; Koumenis, 2006; Jheng et al., 2010; Song et al., 2019). To overcome the ER stress, the eukaryotic cells have developed an adaptive response known as unfolded protein response (UPR) to reduce the load of newly synthesized proteins and misfolded proteins by increased expression of molecular chaperones. Additionally, the proteins that fail to fold are degraded by ER degradation pathway. UPR related ER degradation involves proteasomal degradation and macroautophagy (Fujita et al., 2007; Korolchuk et al., 2010). The UPR consists of three parallel pathways involving 3 different misfolded protein sensors, ATF6, ER-resident integral membrane protein (IRE)1 and proteins kinase RNA (PKR)-like ER kinase (PERK). The precise mechanism of how these three sensors are activated is not well-understood. However, there is a general consensus that the ER luminol chaperone glucose-regulated protein (GRP)78/BIP is normally bound to these sensors to prevent the activation of UPR. When the GRP-78 senses or binds to misfolded proteins, it dissociates from the sensors to activate UPR signaling (Bertolotti et al., 2000; Shen et al., 2002; Kelsen, 2016). Activation of any arm of the UPR increases GRP78 expression via feedback mechanism and this indicates ER stress.

During the viral life cycle, ER stress may arise from the exploitation of ER membrane, accumulation of misfolded proteins, ER calcium imbalance, and depletion of ER membrane during virion release. Many positive strand RNA viruses, including RV have been shown to cause UPR-related ER stress (Su et al., 2002; Tardif et al., 2004; Jheng et al., 2010). Recently RV was shown to increase the expression of GRP78 as early as 6 h post-infection, suggesting RV induces ER stress and this was associated with the activation of PERK and ATF6 arms of UPR, and also an increase in the expression of viral structural protein, VP2 (Song et al., 2019). Moreover, RV 2B protein, which participates in the processing of RV polypeptide during viral replication co-localized with ER membrane. Conversely, RV-stimulated ER stress also induced apoptosis in the cells indicating that ER stress may be required for limiting viral replication. All the work was performed in H1HeLa cells, which are highly susceptible to RV infection; therefore, the consequences of RV-induced ER stress in relevant airway epithelial cells are not known. Enterovirus (EV)71, which is closely related to RV, was also demonstrated to induce ER stress via activation PERK. As observed with RV, EV71 also increased the expression of GRP78. However, overexpression of GRP78 significantly reduced the viral progeny and activation of PERK. These observations suggest that like in RV-infected H1HeLa cells, induction of ER stress and UPR activation by EV1 may be necessary for limiting viral replication; thus, comprising one of the arms of innate antiviral immunity.

### Role of Autophagy in Antiviral Immunity

As described above, ER stress and autophagy are closely related and play an important role in maintaining homeostasis in all eukaryotic cells by mediating the removal of harmful cytoplasmic components, including misfolded proteins, and recycling of the nutrients (Yorimitsu and Klionsky, 2005; Senft and Ronai, 2015). During autophagy, aberrant cytoplasmic constituents and damaged organelles are enveloped in a double membrane vesicles. These vesicles will fuse with the lysosomes to form autophagolysosomes, the components in the autophagolysosomes will be degraded by hydrolases, and the nutrients such as amino acids are recycled (Mijaljica et al., 2011; Feng et al., 2014). In addition to ER stress response, autophagy is also initiated by oxidative stress, nutrient deprivation, and type I IFNs (Ambjorn et al., 2013; Schmeisser et al., 2013; He et al., 2018; O'Grady, 2019). The first step in autophagy is the formation of an isolation membrane at the preautophagosomal site and the membrane originates from ER (Axe et al., 2008). This step is followed by the elongation phase, which involves continuous processing by two ubiquitin-like protein-conjugation systems, the Atg12 and Atg8/LC3 system. Similar to ubiquitination, Atg12 is conjugated to Atg5 by Atg7 and Atg10. Atg7 and Atg3 mediates the conjugation of Atg8 to phosphatidylethanolamine. Both Atg12-Atg5 complex, and Atg8 localize to developing autophagosome and then Atg12-Atg5 complex facilitates lipidation of Atg8/LC3 and directs its subcellular localization. The latter may act as a scaffold protein that supports membrane expansion, and the amount of LC3 protein in the membrane those correlates with the size of autophagosome (Geng and Klionsky, 2008). The mammalian target of rapamycin complex 1 (mTORC1) is the master regulator of autophagy and regulated by nutrient starvation, cellular stressors and growth factors. During nutrient deprivation, AMP-activated kinase (AMPK) induces autophagy by suppressing mTORC1 (Zachari and Ganley, 2017). PI3-kinase/AKT signaling pathway activated by growth factors or cellular stress can also suppress mTOR to induce autophagy. Type I IFNs bind to their receptors leading to Janus kinase and non-receptor tyrosine protein kinase-mediated phosphorylation of insulin receptor substrate 1 and 2, which in turn activate PI3K-AKT. AKT not only inhibits mTOR but also stimulates the expression of a variety of autophagy genes (Mammucari et al., 2008).

Given the degradative function of autophagy, it is conceivable that viral components, viral particles and, host proteins necessary for viral replication can all be captured and removed by autophagy. Thus, autophagy may be an integral part of innate antiviral response. For example, picornaviruses can be detected by galectin 8, which induces autophagy to degrade the viral genome; thus, restricting the viral replication (Staring et al., 2017). P62, a protein that carries cargo to autophagosomes for degradation, binds to Sindbis virus capsid proteins and targets to autophagic degradation (Orvedahl et al., 2010). Induction of autophagy following RV infection has been demonstrated in H1HeLa cells and inhibition of autophagy with 3-methladenine increased viral titer (Jackson et al., 2005; Klein and Jackson, 2011), but the mechanisms underlying how RV subverts autophagy to its own benefit is yet to be determined. Other viruses in the *Picornaviridae* family such as poliovirus and coxsackievirus also subvert autophagy to promote viral replication either by degrading the P62 or by hijacking other host proteins that block galectin 8 (Shi et al., 2013). Similar mechanisms may be used by RV to evade autophagic degradation particularly in susceptible H1HeLa cells (Jackson et al., 2005; Klein and Jackson, 2011). Our on-going studies indicate that RV induces autophagy in airway epithelial and it is not known whether it is beneficial to host or to virus (Sajjan et al., unpublished observations).

Most of the studies to understand the innate immune responses are conducted with either non-epithelial H1HeLa cells or submerged cultures of bronchial epithelial cell lines. Although these cell culture systems are suitable for mechanistic studies, mucociliary-differentiated cultures which closely resemble airway epithelium *in vivo* may reflect the *in vivo* responses more accurately. Unlike submerged cultures, mucociliary-differentiated cell cultures are polarized, and thus separates basal (submucosal side) from the apical (luminal) side with regards to receptor expression and responses to infection. For example, nitric oxide is generated primarily by ciliated cells in response to infection; therefore, effects of nitric oxide in the context of RV infection may not be possible in submerged cultures. CDHR3, a receptor for RV-C is only expressed on ciliated cells; therefore, undifferentiated airway epithelial cells may not be useful in studying responses to RV-C unless the cells are genetically modified (Griggs et al., 2017; Everman et al., 2019). A recent study showed that in mucociliary-differentiated cells, RV induces different vectoriality of release for IFNs (mainly apical), CXCL-10, and IL-17C (mainly basolateral) (Jamieson et al., 2019; Warner et al., 2019). Receptors for IFNs are expressed on the apical surface; therefore, apically released IFNs will readily interact with their receptor to establish antiviral state in the neighboring uninfected cells. On the other hand, basolateral expressed CXCL-10 and IL-17C may play a role in recruitment of T cells, neutrophils, and immature monocytes to submucosa to protect submucosal tissue from the invading virus. CXCL-8, a potent neutrophil chemoattractant is expressed at both apical and basolateral surface after RV infection (Sajjan et al., unpublished observations) and this may create a cytokine gradient for the recruitment of neutrophils into the airway lumen. These studies indicate the advantages of using mucociliary-differentiated cultures in the studies related RV infection.

## Interaction of Airway Epithelial Cells With Hematopoietic Cells During RV Infections

In the lung, airway epithelial cells function in concert with other innate immune cells. There are some studies demonstrating the interaction of airway epithelial cells with recruited hematopoietic cells in the context of RV infection. Although RV bind and enter the monocytes and macrophages, it does not replicate in these cells (Gern et al., 1996). However co-culturing of airway epithelial cells with monocytes increases ICAM-1 expression that promotes RV replication in monocytes, and this was mediated through secreted products by airway epithelial cells (Zhou et al., 2017). In response to RV, airway epithelial cells also secrete CXCL-1, CXCL-8, and CXCL-10 which are potent chemoattractants for neutrophils and immature monocytes, the latter cell type express high amounts of IL-6, CXCL-8, and IFNs in response to RV when co-cultured with epithelial cells (Zhou et al., 2017). Although the role of neutrophils in viral clearance is not known, neutrophils suppress IL-6 and CXCL-8 secretion from monocytes (Tang et al., 2016); therefore, neutrophils may limit RV-induced overt inflammation *in vivo*. Nasal epithelial cells express programmed death ligand (PD)-L1 and PD-L2 in response to RV (Heinecke et al., 2008). These ligands are co-stimulatory molecules and upon ligation to its receptor PD-1 inhibit activation of T and B cells. In a co-culture model of airway epithelial cells and T cells, the airway epithelial cells express PD-L1 and PD-L2 in response to RV, this in turn inhibits T cell activation and expression of IFN-γ, which is indeed necessary for killing virus-infected cells. On the other hand, PD-1 activity is required for the termination of the late phase of allergic inflammation activation (Deppong et al., 2006). Therefore, depending on the specific T cells being engaged, airway epithelial expression of PD-L1 and PD-L2 may be either detrimental or beneficial to host. More recently, bronchial epithelial cells from patients with asthma have been shown to express IL-25 in response to RV, and this can enhance Th2 inflammation (Beale et al., 2014). Secretion of TSLP from airway epithelial cells can drive expansion of type 2 innate lymphoid cell (ILC2) expansion and mucus metaplasia during experimental RV infection in baby mouse model of asthma (Hong et al., 2014; Han et al., 2017). Nasal mucosal epithelial cells express IL-15 following experimental RV infection in humans, which in turn activates CD8+ and natural killer cells to promote the expression of IFN-γ that may contribute to viral clearance (Jayaraman et al., 2014). We have demonstrated that in the absence of TLR2 signaling, RV stimulates exaggerated CXCL-10 expression in airway epithelial cells via activation of IL-33/ST2 signaling axis (Ganesan et al., 2016). COPD airway epithelial cells show heightened CXCL-10 expression in response to RV and this can be attributed to attenuated TLR2 signaling (Schneider et al., 2010; Xander et al., 2019). In a mouse model of COPD RV-induced CXCL-10 promotes excessive recruitment and activation of CD8+ve cells and intermediate macrophages to cause progression of lung disease (Gimenes-Junior et al., 2019a). These observations indicate that airway epithelial cells interact with variety of innate immune cells during RV infection and this can be either beneficial or detrimental to the host depending upon the microenvironment in the lungs.

## Antiviral Immunity is Dysfunctional in Airway Epithelial Cells From Patients With Chronic Obstructive Pulmonary Disease

Epithelium in the airways of patients with COPD often show remodeling, including goblet cell and basal cell metaplasia and sometimes squamous metaplasia (Rock et al., 2010; Gohy et al., 2019). We and others have demonstrated that airway basal/stem cells isolated from COPD bronchi regenerate airway epithelium that is structurally different from the normal in showing basal cell and goblet cell hyperplasia (Schneider et al., 2010; Gohy et al., 2015; Ghosh et al., 2018; Jing et al., 2019). The regenerated COPD airway epithelial cell cultures produce more than normal IL-6, CXCL-1, and CXCL-8, indicating that they may maintain pro-inflammatory phenotype (Schneider et al., 2010). Such differences in regenerated airway epithelial phenotype may arise from the acquired defective repair mechanisms in airway basal cells. Additionally, it may also significantly affect innate immune responses. Consistent with this notion, we found that mucociliary-differentiated COPD airway epithelial cells show exaggerated pro-inflammatory and antiviral responses to RV infection (Schneider et al., 2010). Despite having higher type I and type III IFN responses as well as IFN-stimulated gene expression, COPD cells showed higher viral load. However, both normal and COPD cell cultures completely cleared the virus by 7 days post-infection. Since cigarette smoke is thought to be the primary risk factor for the development of COPD, some studies have used cigarette smoke-exposed airway epithelial cells to investigate the altered innate immune responses to RV. Normal airway epithelial cells acutely exposed to cigarette smoke extract showed downregulation in all the antiviral innate immune responses and an increase in viral load in response to RV infection (Eddleston et al., 2011; Proud et al., 2012; Hudy and Proud, 2013). Similar reduced antiviral responses and an increase in viral load was observed in mucociliary-differentiated cell cultures after acute exposure to RV (Berman et al., 2016). The observed discrepancy may be explained based on the acute vs. years of smoking-induced changes that occur in COPD patients.

**Figure 3:**
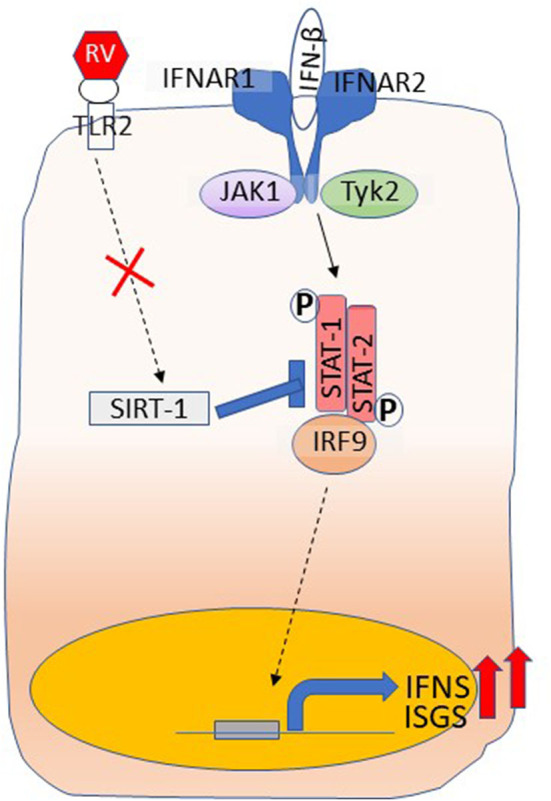
Dysregulated IFN responses in COPD airway epithelial cells. Type I and III IFNs bind to their respective receptors and stimulate the expression of ISGs as well amplify the expression of IFNs through activation of JAK-STAT pathway. RV interaction with TLR2 induces the expression of SIRT-1, which inhibits STAT activation to limit amplification of IFNs and the expression of ISGs and this arm is dysregulated in COPD. Shown in the figure is binding of IFN-β to its receptors, IFNAR1 and IFNAR2. Interaction of IFN-λs with its receptors IFNLR1 and IL10R2 activates JAK-STAT signaling similar to IFN-β.

One of the mechanisms underlying higher IFN responses is due to reduced expression of SIRT-1, which is required for limiting the amplification phase of IFN expression that occurs as a result of activation of IFN receptor signaling (Xander et al., 2019) (Figure 3). Furthermore, SIRT-1 expression following RV infection requires functional TLR2, and the TLR2 signaling pathway is dysregulated in COPD airway epithelial cells. Another important feature that we observed was at later stages of RV infection that is at 14 days post-infection, COPD, but not normal cell cultures showed further increase in the number of goblet cells and expression of mucin genes (Jing et al., 2019). This was due to persistent activation of NOTCH signaling in COPD cell cultures following RV infection. Interestingly, at this stage, there was no detectable RV indicating that RV infection may induce changes that are sustained in COPD cells even after the virus cleared. This may be due to defective anti-inflammatory or repair pathways, and needs further investigation.

## Innate Immune Responses of Bronchial Epithelial Cells From Patients With Asthma and CF to RV Infection

Airway epithelium in patients with asthma and cystic fibrosis (CF) also show remodeling with goblet cell metaplasia and respond abnormally to RV infection. Based on the outcome of *in vitro* and *in vivo* studies, it is thought that RV may enhance Th2 cytokines via the expression of TSLP and IL-25 derived from airway epithelial cells; thus, exacerbating the lung disease in patients with asthma (Kato et al., 2007; Uller et al., 2010; Han et al., 2017). There are also conflicting data with regards to antiviral IFN responses to RV in patients with asthma. Some studies demonstrated that primary airway epithelial cells from asthma are defective in mounting antiviral IFNs, particularly type III IFNs expression in response to RV (Wark et al., 2005; Contoli et al., 2006). Later on, other studies showed no difference in IFN responses to RV infection between the normal and asthmatic airway epithelial cells (Bochkov et al., 2010; Bai et al., 2015; Ravi et al., 2019). As observed in COPD cells, airway epithelial cells from asthmatic subjects showed higher expression of certain genes, such as ICAM-1 Inhibin beta, LOXL2A, IL-6, and IL-1β at basal levels (Bochkov et al., 2010). Following RV infection, although there was no difference in the viral clearance between the normal and asthmatic airway epithelial cells, the latter showed some differentially regulated genes, which include CCL5, CXCL10, and CX3CL1, MUC5AC, CDHR3 (Bai et al., 2015). Moreover, bronchial cells from experimentally-infected asthmatic patients showed higher IFNs, which correlated with higher viral load in the nasopharyngeal cavity (Ravi et al., 2019). The observed differences between these studies may be due to polymorphisms that have been recently reported to affect innate immune responses to RV infection. Polymorphism in chromosome 17q21, which positively correlates with orosomucoid like 3 (ORMDL3) expression confers the major genetic susceptibility not only to onset of childhood asthma, but also to RV infection-associated wheezing (Moffatt et al., 2007; Caliskan et al., 2013). ORMDL3 inhibits serine palmitoyltransferase, and the expression of ORMDL3 in epithelial cells was associated with a reduction in RV-induced ER stress and IFN responses and concomitant increase in viral load (Liu et al., 2020). While the pharmocologic induction of ER stress reduced viral replication and increased IFN expression in ORMDL3 expressing cells, inhibition of serine palmitoyltransferase negated the effects of ORMDL3 knockdown. These results indicate that ORMDL3 may negatively regulate ER stress- and sphingolipid-associated antiviral immunity. However, how sphingolipid regulates antiviral immunity is not known.

IFN responses to RV infection in CF airway epithelial cells is also controversial. While one study demonstrated reduced IFN responses to RV infection (Vareille et al., 2012), we and others have demonstrated that RV induces higher than normal IFN responses in mucociliary-differentiated CF bronchial epithelial cells (Chattoraj et al., 2011; Dauletbaev et al., 2015). We further demonstrated that prior infection with *Pseudomonas aeruginosa* which causes chronic infection in CF airways, reduces RV-induced IFN responses in CF but not in normal airway epithelial cells, and this is due to enhanced oxidative stress.

## Conclusions

Conductive airway epithelial cells, the primary target of RV, not only function as a physical barrier but also plays a pivotal role in mounting appropriate innate immune responses. Airway epithelial cells are equipped with layers of antiviral immune mechanisms. Nitric oxide that is generated immediately after an infection has potent antiviral activity and modulates inflammatory cytokine expression to avoid overt inflammation. Type I and type III IFNs-stimulated genes, in addition to interfering with viral replication also limit virus-induced pro-inflammatory cytokines to prevent excessive inflammation. Although controversial, some studies correlated reduced IFN expression with higher viral load in patients with asthma. Even though, prophylactic treatment with IFNs limits influenza viral replication *in vitro* (Watson et al., 2020 #215), it had no significant effect on asthma symptoms in a small clinical trial (Djukanovic et al., 2014 #214). In contrast, overexpression of these cytokines may trigger unwanted inflammation. Therefore, the airway epithelial cells have evolved to overcome this effect by expressing anti-inflammatory or inhibitors of signaling pathways. In the context of RV infection, TLR2 signaling appears to play an important role in limiting overexpression of RV-induced IFNs via SIRT-1 expression. SIRT-1 inhibits STAT-1/2, which occurs during the amplification of IFNs via interaction of IFNs with its receptors. This mechanism is dysregulated in COPD airway epithelial cells, which show heightened expression of IFNs in response to RV. Airway epithelial cells-derived chemokines recruit a variety of innate immune cells, which detect and clear virus-infected epithelial cells and apoptotic cells, establish antiviral state and resolve the acute inflammation caused by RV. If these protective innate immune responses of airway epithelial cells to RV are not tightly regulated, it can lead to excessive or persistent inflammation due to continued recruitment and activation of T cells, macrophages and neutrophils. The fact that airway epithelial cells express number of molecules, including DUSP, TOLLIP, IRAK-M, which negatively regulate MAPK and TLR pathways indicate that under normal situation, airway epithelial cells can balance pro-inflammatory to anti-inflammatory responses to RV. Although, ER stress and autophagy play important role in limiting replication in RV-infected H1 HeLa cells, whether these processes affect viral replication or expression of pro-inflammatory cytokines in airway epithelial cells is yet to be determined. It is also not known how ER stress and autophagy affect viral clearance or virus-induced inflammation in patients with chronic lung disease who already show increase in ER stress and autophagy.

Despite these new advances in the field of innate immune responses to RV, mechanisms underlying the RV-associated exacerbations in patients with chronic lung diseases are not completely understood. While COPD patients often show Th1 inflammation, majority of the patients with asthma show Th2 inflammation in response to viral infections. Therefore, the mechanisms underlying RV-associated inflammation in asthma and COPD may be very different and this should be considered while translating the findings to develop new therapies.

## Author Contributions

HG wrote description on rhinovirus. CR and ME conducted literature search and provided description on autophagy and ER stress. US wrote the manuscript.

## Conflict of Interest

The authors declare that the research was conducted in the absence of any commercial or financial relationships that could be construed as a potential conflict of interest.
